# Functional Neurological Symptom Disorder Manifesting as Auditory Verbal Agnosia in a 19-Year-Old Patient

**DOI:** 10.7759/cureus.27930

**Published:** 2022-08-12

**Authors:** Suzanna Kitten, Neel D Jani, Daniel A Llano

**Affiliations:** 1 Psychiatry, Carle Illinois College of Medicine, Urbana, USA; 2 Psychiatry, Carle Foundation Hospital, Urbana, USA; 3 Internal Medicine, Carle Foundation Hospital, Urbana, USA; 4 Neurology, Carle Foundation Hospital, Urbana, USA; 5 Molecular and Integrative Physiology, University of Illinois at Urbana-Champaign, Urbana, USA

**Keywords:** post-traumatic stress disorder, conflict, conversion reaction, conversion neurosis, auditory verbal agnosia, conversion disorder, functional neurological symptom disorder

## Abstract

Functional neurological symptom disorder (FNSD), otherwise known as conversion disorder (CD), is a condition in which neurological deficits cannot solely be explained by medical pathology. Auditory verbal agnosia (AVA) is the inability to understand speech. While these two conditions are well-documented independently, a case of FNSD manifesting as AVA has not been previously reported. We present a 19-year-old patient, with a history complicated by congenital cardiomyopathy resulting in chronic heart failure with reduced ejection fraction and alpha-thalassemia, who demonstrated these symptoms. This case details the effectiveness of a multi-pronged treatment approach that was implemented over several years, eventually leading to the resolution of the conversion symptoms.

## Introduction

Functional neurological symptom disorder (FNSD), also known as conversion disorder (CD), is a condition with etiology that is not precisely known. Characterized by neurological deficits driven by psychological factors, it is typically viewed from a psychodynamic perspective, where a subconscious conflict between an individual’s desire and conscience manifests as a physical symptom [1]. Recent imaging and behavioral data have implicated disruptions in neural circuits governing emotional processing, agency, and self-monitoring [2-6]. Auditory verbal agnosia (AVA) is the inability to comprehend verbal speech, but with retention of other forms of comprehension, such as written information [7].

We describe a unique case of a 19-year-old female who demonstrated symptoms of AVA as a result of FNSD. While both are well-explored conditions, to the authors’ knowledge, there are no cases in the literature describing FNSD manifesting as AVA.

## Case presentation

A 19-year-old right-handed female patient was first seen in May 2015. She presented with extreme cognitive decline and an inability to understand spoken language. While she understood written language, she did not respond to speech. During appointments, the patient would not speak except to ask her mother, “Huh, what did she say?” The mother would use hand gestures and writing to communicate with her daughter who would then verbally reply.

The patient was born at full term without complications; however, speech issues became apparent in kindergarten when she was diagnosed with a processing deficit. During elementary school, the patient developed anxiety. At age 16, Full Scale Intelligence Quotient (FSIQ) testing was performed with a score of 68. This finding, coupled with a severely impaired level of adaptive functioning, resulted in a diagnosis of mild intellectual disability (ID). Placed in special education classes, the patient began to experience depression, paranoia, and suicidal ideation during her sophomore year of high school after undergoing substantial bullying. She was psychiatrically hospitalized for two days during this time and started on venlafaxine titrated to 112.5 mg/day. At age 18, the patient scored 18/30 on the Mini-Mental State Examination (MMSE).

Following the initial consultation, a laboratory workup was obtained to rule out an organic cause for the patient’s psychiatric and cognitive decline. Urine copper studies, porphyria, rapid plasma reagin (RPR), antinuclear antibody (ANA), Lyme, venereal disease research laboratory (VDRL), and HIV testing were negative; ceruloplasmin, vitamin B12, homocysteine, thiamine, erythrocyte sedimentation rate (ESR), and creatine kinase (CK) values were within the normal range. A multiple sclerosis panel and CSF analysis for paraneoplastic autoantibodies were negative. A brain MRI showed a 1-mm non-enhancing T2 hyperintensity in the right subinsular region but was otherwise normal. Audiology testing indicated normal bilateral hearing sensitivity. EEG did not show any paroxysmal activity, spikes, or sharp waves.

Neuropsychological testing was conducted in an effort to better characterize the patient’s symptoms. Using the Peabody Picture Vocabulary Test (PPVT-4), her receptive vocabulary was determined to be that of a six-year-old. An expressive vocabulary test (EVT-2) revealed her expressive vocabulary to be equal to a child of five years and seven months old, showing a significant decline from prior functioning.

The negative serological workup, as well as the patient’s clinical symptoms and history of adolescent trauma, led to a diagnosis of FNSD with AVA. Namely, the patient’s trauma from bullying likely led to an unconscious conflict that manifested itself as AVA with the inability to hear what others were saying unless interpreted by a safe source, her mother [1].

Early on, it was suspected that an underlying co-morbid post-traumatic stress disorder (PTSD) was contributing to the patient’s symptoms due to the intense bullying she experienced in high school. At age 19, her PTSD checklist (PCL-5) score was 39. This checklist has a cut-off score of 32 in specialty Veterans Affairs (VA) clinics for PTSD diagnosis [1]. By the time the patient turned 22, she began experiencing racing thoughts, excessive talking, increased pacing, and limitless energy despite only four to five hours of sleep. The onset of these symptoms suggested an underlying co-morbid bipolar disorder complicating her diagnosis. Epileptic encephalopathies such as Landau-Kleffner syndrome were considered but are unlikely given the absence of typical EEG characteristics [7].

Stabilizing this patient from a psychiatric perspective proved challenging. Ineffective trials of olanzapine (10 mg/day), haloperidol (5 mg/day), aripiprazole (10 mg/day), quetiapine (up to 600 mg/day), paroxetine (up to 5 mg/day) [7], lamotrigine (up to 200 mg/bid), and escitalopram (up to 10 mg/day) were prescribed for the patient’s worsening mood and PTSD symptoms.

At age 22, with the start of symptoms suggestive of resistant bipolar disorder, clozapine, titrated to 300 mg/day, was initiated. As the clozapine produced over-sedating side effects, including drooling and flattening, the dose was reduced to 50/150 mg/bid [8].

In addition to pharmacotherapy, the patient received psychotherapy to improve her socialization and communication skills. She met with a therapist every two weeks and received eye movement desensitization and reprocessing treatment, as well as speech therapy. The speech therapy enabled her to not only lip-read but repeat the phrase back to herself and then answer. Concomitantly, she obtained a job at a church nursery and enrolled in classes at a local college.

During a cardiac workup at age 23, the patient was found to have probable congenital cardiomyopathy resulting in chronic heart failure with an ejection fraction (EF) as low as 30-35%, as well as alpha thalassemia. She was subsequently transitioned off the clozapine and onto paliperidone (3 mg/day) for her severe bipolar disorder with psychotic features [9]. She continued on duloxetine (30 mg/day) for anxiety and suvorexant (20 mg/qhs) and ramelteon (8 mg/qhs) for severe insomnia. Her FNSD symptoms are now at almost complete resolution. While the patient can communicate largely normally in-person by reading lips, she still has some perceived difficulty hearing when not communicating in person. As such, certain activities like talking over the phone sometimes are more difficult.

## Discussion

While FNSD can be explained from a psychodynamic standpoint, research using functional imaging indicates biological mechanisms may be at play. For example, disturbances in neural networks between the frontal cortex and limbic system can lead to inhibitory signals from the basal ganglia that affect signals to the primary motor cortex [8]. In addition, altered functional connectivity between limbic and motor regions has been uncovered in patients with functional movement disorders using functional magnetic resonance imaging [9], suggesting that involuntary movements in these patients may be triggered, or at least modulated, by emotional factors. In the case of AVA, given that organic AVA is often the result of lesions in Heschl’s gyrus in the primary auditory cortex [10], it is possible that functional AVA may be related to abnormal functional connectivity between limbic regions and Heschl’s gyrus. Recent work in animal models has established a direct projection from the amygdala to the primary auditory cortex and that this pathway is involved in linking auditory and emotional signals [11]. This pathway shows plasticity such that emotional trauma may modify its activity and thus modify the responsiveness of the auditory cortex to verbal inputs. Although the Diagnostic and Statistical Manual of Mental Disorders (DSM-5) [1] recently eliminated prior trauma as a diagnostic criterion for FNSD, stressors have historically been key factors in both cause and diagnosis [12]. Thus, it is possible in our case that early childhood verbal trauma (i.e., bullying) may have modified such limbic-auditory connections, leading to this patient’s highly selective responsiveness to particular auditory inputs as a young adult. Future work will be needed to identify direct limbic-to-auditory inputs in humans and if these pathways are altered by early childhood trauma.

## Conclusions

This is a case of a patient who exhibited symptoms of FNSD with AVA. Her adolescent trauma of bullying likely led to an unconscious conflict that manifested as AVA. Undoubtedly, the patient’s PTSD contributed to the development of FNSD. However, more severe mental illness (bipolar disorder with psychotic features) revealed itself at age 22, which coincides with a typical age of onset in females. Additionally, this case was confounded by her apparent cognitive decline from baseline, as well as severe medical complications (congenital cardiomyopathy and alpha thalassemia). While addressing the underlying PTSD and bipolar disorder was integral in this patient’s treatment, psychotherapy combined with speech and social interaction therapies proved beneficial components in improving her quality of life. This underscores the importance of utilizing a holistic approach when treating patients with FNSD.
